# Earliest modern human-like hand bone from a new >1.84-million-year-old site at Olduvai in Tanzania

**DOI:** 10.1038/ncomms8987

**Published:** 2015-08-18

**Authors:** Manuel Domínguez-Rodrigo, Travis Rayne Pickering, Sergio Almécija, Jason L. Heaton, Enrique Baquedano, Audax Mabulla, David Uribelarrea

**Affiliations:** 1 IDEA (Instituto de Evolución en África), Museo de los Orígenes, Plaza de San Andrés 2, 28005 Madrid, Spain; 2 Department of Prehistory, Complutense University, Prof. Aranguren s/n, 28040 Madrid, Spain; 3 Department of Anthropology, University of Wisconsin-Madison, 1180 Observatory Drive, Madison, Wisconsin 53706, USA; 4 Evolutionary Studies Institute, University of the Witwatersrand, WITS, 2050 Johannesburg, South Africa; 5 Plio-Pleistocene Palaeontology Section, Department of Vertebrates, Ditsong National Museum of Natural History (Transvaal Museum), Pretoria 0002, South Africa; 6 Center for the Advanced Study of Human Paleobiology, Department of Anthropology, The George Washington University, Washington, District of Columbia 20052, USA; 7 Department of Anatomical Sciences, Stony Brook University, Stony Brook, New York 11794-8081, USA; 8 Institut Català de Paleontologia Miquel Crusafont (ICP), Universitat Autònoma de Barcelona, Edifici Z (ICTA-ICP), campus de la UAB, c/ de les Columnes, s/n, 08193 Cerdanyola del Vallès (Barcelona), Spain; 9 Department of Biology, Birmingham-Southern College, Birmingham, Alabama 35254, USA; 10 Museo Arqueológico Regional, Plaza de las Bernardas s/n, 28801 Alcalá de Henares, Madrid, Spain; 11 Archaeology Unit, University of Dar es Salaam, Dar es Salaam, PO Box 35050 Tanzania; 12 Department of Geodynamics, Complutense University, c/ José Antonio Novás 12, 28040 Madrid, Spain

## Abstract

Modern humans are characterized by specialized hand morphology that is associated with advanced manipulative skills. Thus, there is important debate in paleoanthropology about the possible cause–effect relationship of this modern human-like (MHL) hand anatomy, its associated grips and the invention and use of stone tools by early hominins. Here we describe and analyse Olduvai Hominin (OH) 86, a manual proximal phalanx from the recently discovered >1.84-million-year-old (Ma) Philip Tobias Korongo (PTK) site at Olduvai Gorge (Tanzania). OH 86 represents the earliest MHL hand bone in the fossil record, of a size and shape that differs not only from all australopiths, but also from the phalangeal bones of the penecontemporaneous and geographically proximate OH 7 partial hand skeleton (part of the *Homo habilis* holotype). The discovery of OH 86 suggests that a hominin with a more MHL postcranium co-existed with *Paranthropus boisei* and *Homo habilis* at Olduvai during Bed I times.

## Abstract (web summary)

The homin fossil record reveals a complex pattern of hand evolution. Here, the authors describe a phalanx of a >1.84-million-year-old unidentified hominin, which represents the earliest modern human like hand bone in the fossil record.

## Graphical abstract

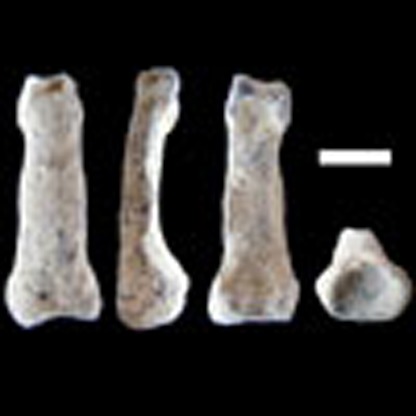

The sophisticated manipulative skills that characterize modern humans (*Homo sapiens*) have been related to our derived hand morphology (for example, long thumb relative to fingers, robust joints and hypertrophic pollical muscles)^1^,^2^,^3^, which allows for combined power and—uniquely among hominoids—pad-to-pad precision grasping^1^,^4^. Commonly, these modern human-like (MHL) grips and linked MHL manual anatomy are interpreted as specific adaptations for the efficient manufacture and use of stone tools, another purported hallmark of ‘humanness' (for example, refs 2, 3, 5, 6). However, as the hominin fossil record expands, a complex pattern of hand evolution is now apparent: the hand bones of some Pliocene australopiths are actually more MHL than are those of other, more recent Pleistocene hominins, suggesting that systematic manufacture and use of stone tools could well have emerged in hominins that already possessed skilful hands^4^,^7^,^8^,^9^. The earliest tools, associated functionally with butchered ungulate bones, are dated to 2.6-million-year-old (Ma; refs 10, 11) and, even older, 3.3-Ma lithic artefacts have also been recently announced^12^. Thus, although the fossil record indicates that Pliocene hominins possessed overall MHL hand proportions and probably advanced manipulatory skills, most available postcranial evidence of this period (including hand bones) also reflects adaptations consistent with habitual engagement in arboreal locomotion^13^,^14^,^15^,^16^ (although see refs 17, 18). Indeed, it is only <2 Ma that key regions of the hominin postcranial skeleton exhibit fully MHL morphologies^19^,^20^, which may indicate an adaptive commitment to a terrestrial MHL lifestyle. The new Olduvai Hominin (OH) 86 manual proximal phalanx, described here and dated to >1.8 Ma (refs 21, 22, 23), enriches our understanding of this critical period of transition to a more MHL body form in our ancestors.

Incipient expressions of MHL hand morphology can be traced to the very earliest phase of hominin evolution. *Orrorin tugenensis* (Kenya), at ∼6.0 Ma, exhibited a pollical distal phalanx with distinctive anatomy, including a proximal palmar fossa/gable ridge complex, an ungual fossa and a mediolaterally wide apical tuft^8^,^24^. These features indicate that *O. tugenensis* possessed an attachment site for a long tendon of a pollical flexor muscle and are also related to specific adaptations for MHL pad-to-pad precision grasping^8^,^25^. Based on its original description^26^, it is unclear whether 4.4-Ma *Ardipithecus ramidus* engaged in MHL pad-to-pad precision grasping, although a recent study indicates that this was probably unlikely^27^. However, the pollical distal phalanges of *Australopithecus afarensis* (3.6–2.9 Ma), *Au. africanus* (∼3.0–2.0 Ma) and *Au. sediba* (1.98 Ma), similar to those of *O. tugenensis*, also show morphology indicative of MHL pad-to-pad grasping^8^,^14^,^25^,^28^,^29^. Beyond indirect evidence of MHL grips in some hominin species, the reconstructed hand skeleton of *Au. afarensis*^4^,^29^ (although see ref. 30), and the associated hand of *Au. sediba*^31^, both exhibit a long thumb relative to the fingers, the main requirement for a pad-to-pad contact (and thus MHL precision grasping). This reinforces the hypothesis that australopiths were able to engage in enhanced, refined MHL manipulation either before or at the advent of systematic stone tool making^4^,^6^,^7^,^8^,^9^,^12^,^29^. Indeed, even though the pollical phalanges of these australopiths possess plesiomorphic, dorsopalmarly thick apical tufts, they are, in general, still more MHL than are the phalanges of some other fossil hominin taxa^8^,^14^,^28^.

Yet, despite the impressive range of interspecific morphological variability evinced in the early portion of the hominin fossil record, and the unavoidable conclusion that several premodern taxa probably possessed at least some capability for MHL precision gripping, there remain some important differences between the hand skeletons of early hominins and those of modern humans. For example, like earlier, more plesiomorphic forms, such as *O. tugenesis, Ar. ramidus* and several australopiths, the ∼1.84-Ma (ref. 22) OH 7 hand skeleton^32^ (part of the *H. habilis* holotype^33^) possesses proximal and intermediate phalanges that show marked palmar curvature, mediolaterally expanded diaphyses and strong flexor apparatuses—all features that are commonly associated with regular arboreal locomotion^1^,^7^,^14^,^32^. Further, the pollical distal phalanx of OH 7 also lacks a definitive insertion site for the long flexor tendon and other features that are related to pad-to-pad precision gripping^8^. Indeed, the overall morphology of the OH 7 phalanges is reminiscent of that of some of its ∼2.0–1.0-Ma homologues from the South African site of Swartkrans that do not fit a human pattern^7^. Because the dentognathic remains of *Paranthropus robustus* dominate the taxonomically mixed hominin fossil samples from the Swartkrans Formation, the Swartkrans hominin phalanges that do not fit a MHL pattern were assigned to this species by Susman^34^,^35^. For this reason, Moyà-solà *et al.*^7^ hypothesized that the OH 7 hand, with phalanges that are *less* MHL than those of some earlier australopiths, might derive from *Paranthropus* rather than from *Homo.* The best test of this hypothesis will obviously be to discover dentognathic remains of *Paranthropus* in clear and exclusive association with phalanges (and other hand bones). Obviously, hand bones (phalanges in this case) of *Paranthropus* in clear association with dentognathic remains will be necessary to test this hypothesis.

Against this complex anatomical and functional backdrop, we analyse here the newly discovered complete proximal phalanx OH 86, which, based on morphometric and qualitative evidence, most likely derives from a left ray V. Our analyses—comparing OH 86 to samples of manual proximal phalanges of modern humans and other African catarrhines, as well as to fossil hominin manual proximal phalanges that have been attributed to ray V—reveal that the new Olduvai fossil represents the earliest known hominin hand bone (>1.84 Ma) with MHL appearance. Our results, along with the archaeological record, reveal that instead of following an orderly diachronic trend, eventually culminating in the modern human condition, some ‘primitive' hand bone morphologies persisted side-by-side with MHL hand bone morphologies well after the first appearance of stone tools and zooarchaeological evidence of their use for butchery by at least 2.6 Ma (refs 10, 11). Although other regions of the hand and the skeleton are necessary to provide the most complete picture of the body plan of the hominin present in the new Philip Tobias Korongo (PTK) site at Olduvai, combined, the available data not only highlight the taxonomic and functional diversity of hominins during the Pliocene and early Pleistocene (for example, refs 15, 16, 18) but they also add to an emerging appreciation that an incipient MHL postcranium developed very early in hominin evolution (for example, refs 19, 20).

## Results

### Location

**Figure 1:**
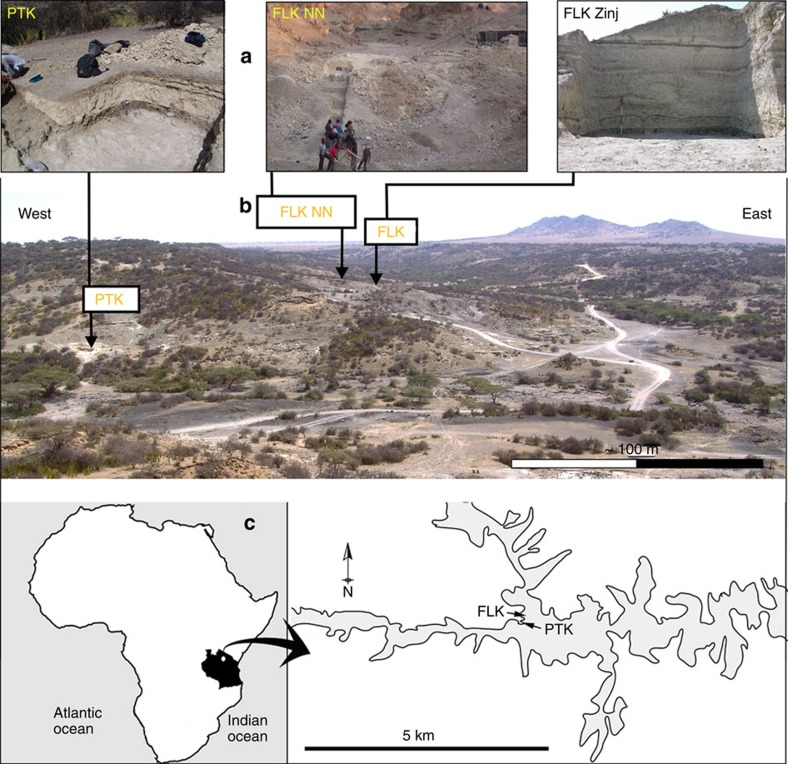
Geographic location of the ‘PTK' site. The location of the new PTK site (from which the OH 86 proximal phalanx was excavated) compared with two other major and penecontemporaneous Middle Bed I (Olduvai Formation) sites of FLK 22 *Zinjanthropu*s and FLK NN that also occur near the junction of the Main and Side Gorges. (**a**) Informal views of excavations at each site. (**b**) Relationship of the sites in aerial view; lower middle image=a panoramic view of the Gorge looking north, with PTK indicated. (**c**) Political map of Africa with Tanzania highlighted in black and the approximate location of Olduvai Gorge represented by white dot and a schematic plan view of sites near the junction of the Main and Side Gorges.

The PTK site was discovered in 2012 by The Olduvai Paleoanthropology and Paleoecology Project (TOPPP) at the junction of the main and secondary branches of Olduvai Gorge (Tanzania; Fig. 1 and Supplementary Fig. 1). The site is situated ∼500 m south of the well-known FLK 22 *Zinjanthropus* (FLK 22 Zinj) site and, to date, it is known to comprise three distinct archaeological levels (Supplementary Fig. 2). Two of these levels, corresponding to what has been defined as ‘upper Zinj' and ‘lower Zinj'^21^, occur in the same clay stratum as the FLK 22 Zinj level, underlying volcanic Tuff IC, dated by ^40^Ar/^39^Ar to 1.832+0.003 Ma (ref. 22). PTK's third archaeological level underlies the Zinj clay, within the tuffaceous layer known as the ‘Chapati Tuff'^23^, and corresponds stratigraphically to the top of the Olduvai Bed I archaeological level designated as FLK NN 2. TOPPP's 2014 excavation of this third level at PTK yielded abundant Mode I stone artefacts and a large faunal assemblage, which includes the MHL hominin proximal phalanx OH 86, described here.

### Specimen identification and anatomical description

**Figure 2:**
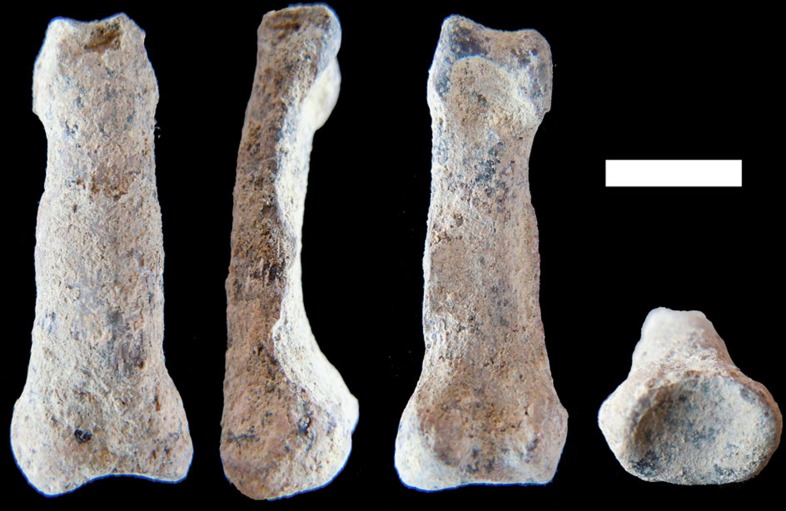
OH 86 views. The OH 86 hominin manual proximal phalanx in (from left to right) dorsal, lateral, palmar (distal is top for each) and proximal views. Scale bar, 1 cm.

OH 86 is a complete manual proximal phalanx that is nearly entirely encased in a very thin layer of the carbonated tuffaceous silt from which it derives (that is, the ‘Chapati Tuff'; Fig. 2). Although the areal spread of this concretion on OH 86 is encompassing, its submillimetre thinness guarantees little impact on the gross measurements that we derived on the specimen and analyse and discuss in this study. Basic osteometrics of OH 86 are listed in Supplementary Table 1. On the basis of several lines of morphometric evidence (see below), as well as qualitative features, we assign OH 86 to the fifth ray of the left hand.

Applying published qualitative criteria^36^—including asymmetry of the specimen's flexor ridges and distal trochlea, as well as the orientation of the latter, and its base's palmar outline—OH 86 compares most favourably to a modern human manual proximal phalanx from ray V. Quantitative data—including head mediolateral width/base mediolateral width ratio=0.72, base mediolateral width/overall superoinferior length ratio=0.39—corroborate this qualitative diagnosis. Last, we tested this corresponding qualitative/quantitative ray assignment by conducting a discriminant function analysis of OH 86 and a comparative sample composed of modern human proximal phalanges from rays II and V (which tend to be more similar to each other because of asymmetries caused by muscle insertions, among others). This analysis confirms the results of the initial qualitative and quantitative tests, also indicating that OH 86 most probably derives from a fifth ray (with a probability six times more likely than ray II, using the seven shape variables, and with a probability 10 times more likely than ray II, using the seven raw dimensions; Supplementary Fig. 3).

Assuming that the assignment of OH 86 as a fifth proximal phalanx is correct, it must also derive from a left hand on the basis of the pattern of asymmetry of the distal condyles: in palmar and dorsal views, the presumed radial condyle projects more inferiorly than does the presumed ulnar condyle. Further, the putative ulnar basal tubercle (insertion for the hypothenar muscles) is larger and protrudes more ulnarly and proximally than does the radial basal tubercle.

*Mosimann shape ratios.* The overall size (as approximated by the geometric mean, GM) of OH 86 is within the range of modern humans and chimpanzees (Supplementary Fig. 4a), as it is the case of other hominins except *Au. sediba* (below the human range). In terms of relative length, OH 86 is in the midrange of humans and the upper range of gorillas, but below chimpanzees and monkeys (Supplementary Fig. 4b). OH 86 exhibits a MHL, dorsopalmarly short trochlea, although this value also overlaps with the lowermost range of African apes (Supplementary Fig. 4c). No trend is evident in mediolateral trochlear width, although it is worth noting that the trochlear proportions of OH 86 are virtually identical to those of the fossil Qafzeh 9 (*H. sapiens*; Supplementary Fig. 4d). With regard to midshaft dimensions, OH 86 is dorsopalmarly short (but still overlaps with the modern human outlier range; Supplementary Fig. 4e), and as a consequence it is also mediolaterally wider than are the midshafts of the proximal phalanges of other hominins (Supplementary Fig. 4f). Extant taxa exhibit similar values of relative basal dorsopalmar height as does OH 86 (Supplementary Fig. 4g), although *Pan* clearly stands out in its having relatively higher bases (but still overlapping with the remaining sample). In this respect, although all hominins fall within the modern human variation, a clear trend is evident: fossil *Homo* and OH 86 show very similar values, in the low interquartile range of *H. sapiens*, whereas all australopiths are in the upper interquartile range. Last, with regard to relative basal breadth (Supplementary Fig. 4h), modern humans (and cercopithecid monkeys) possess wider bases than do African apes. Pliocene australopiths fall in the African ape range, whereas the early Pleistocene *Au. sediba*, fossil *Homo* and OH 86 all exhibit values within the human range.

**Figure 3:**
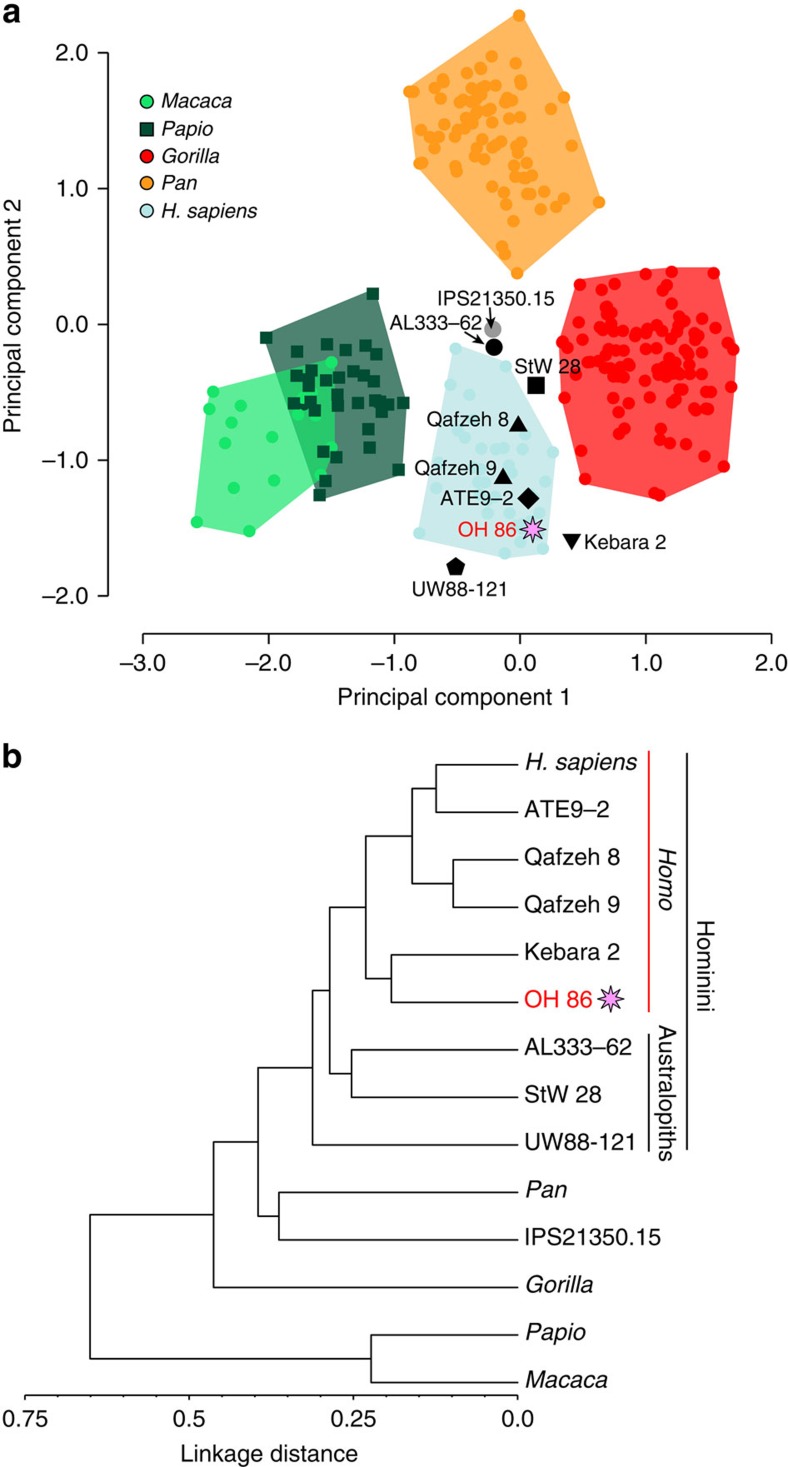
The form of the human proximal phalanx. (**a**) Plot showing the two major axes of proximal phalanx V form variation (that is, shape and size space). Major taxonomic groups can be distinguished (using convex hulls); OH 86 is the earliest fossil specimen *within* the human variation. (**b**) UPGMA cluster analysis summarizing eight dimensions of phalangeal form space: OH 86 is the oldest specimen within the *Homo* cluster. The cophenetic correlation coefficient is high (0.8681), indicating that the dendrogram is faithfully preserving the pairwise distances between the original dimensions. (These analyses exclude OH 7 because this hand skeleton does not preserve complete proximal phalanges^32^.)

**Figure 4:**
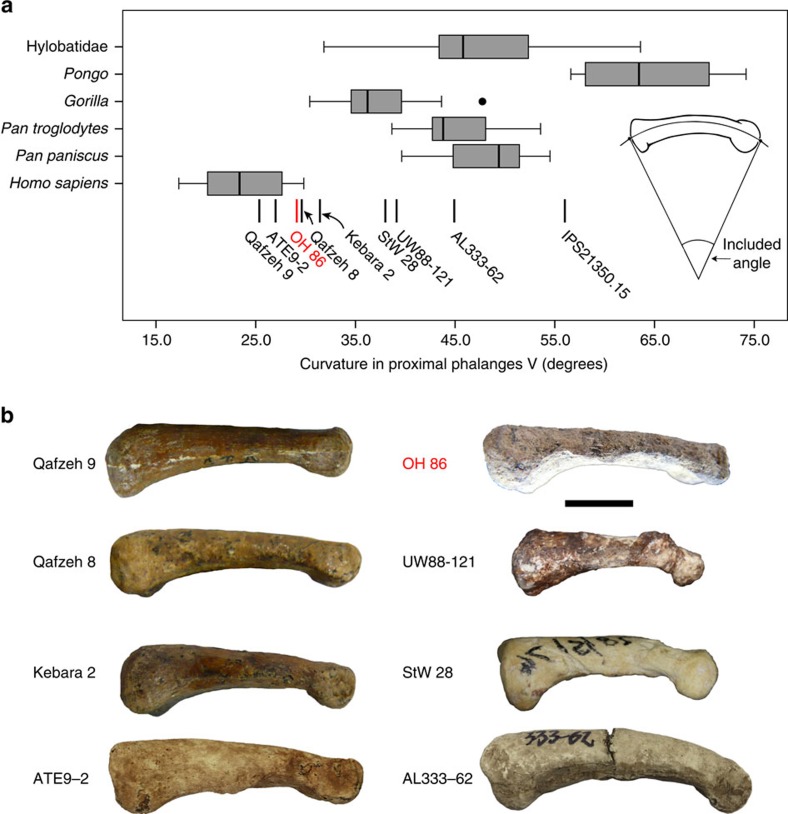
Phalangeal curvature in extant and fossil hominoids. (**a**) Included angle values (in degrees) in a modern and fossil sample of fifth proximal phalanges. OH 86 is (exclusively) within the modern human variation (distinct from australopiths). Boxes represent 25th and 75th percentiles, centreline is the median, whiskers represent non-outlier range and the dot is an outlier. Samples for each boxplot are *Homo sapiens* (*n*=36), *Pan paniscus* (*n*=8), *Pan troglodytes* (*n*=16), *Gorilla* (*n*=22), *Pongo* (*n*=16) and Hylobatidae (*n*=22). (**b**) The fossil hominin specimens analysed in this study are compared in lateral view. All pictures were taken from the originals with the exception of AL333-62 (cast) and ATE9-2 (modified from the literature^56^; Supplementary Table 4). Scale bar, 1 cm.

When all these dimensions of proximal phalanx form variation (that is, the seven Mosimann shape ratios and the associated GM) are summarized by means of a principal components (PCA; Fig. 3a) and a cluster (Fig. 3b) analyses, the closest form affinities of OH 86 are revealed to be with *Homo*. In fact, based solely on the two major axes of form variation, our PCA (Supplementary Fig. 4 and Supplementary Table 5) clearly separates *H. sapiens* from *Pan*, *Gorilla* and cercopithecoid monkeys (Fig. 3a). Further, although all fossils exhibit their closest form affinities to *H. sapiens*, OH 86 is the oldest hominin phalanx *within* the modern human form space (as represented by the two first axes, accounting for 90.1% of total form variation). In addition, when all dimensions of phalangeal form are summarized using an unweighted pair group method with arithmetic mean (UPGMA) dendrogram based on group centroids (Supplementary Fig. 4), it reveals a ‘*Homo*' cluster nested within the ‘hominin' group—with OH 86 being the oldest fossil in the sample placed within this ‘*Homo*' cluster (Fig. 3b). In sum, modern human phalangeal form (Fig. 4 and Supplementary Table 5) is characterized by moderate relative total proximodistal length, midshaft mediolateral robusticity and overall size (that is, intermediate values of PC1, similar to *Pan*) in combination with a mediolaterally wide and dorsopalmarly short trochlea and base (which, together with their shorter lengths, differentiate human and *Pan* proximal phalanges). More specifically, the bases of *Homo* proximal phalanges are mediolaterally wider and dorsopalmarly shorter than are those of australopiths (Supplementary Fig. 4g,h). In addition, on the basis of trochlear shape, the intermediate phalanges of australopiths—as well as those of OH 7—are clearly distinct from those of extant and fossil *Homo*^7^. This previous finding regarding manual intermediate phalanges corresponds to our independent analyses of complete manual proximal phalanges, indicating together that modern human phalangeal morphology can be accurately characterized quantitatively. Importantly, OH 7 does *not* conform to the modern human characterization, even though it is penecontemporaneous with the MHL OH 86.

*Phalangeal curvature.* From functional and evolutionary perspectives, it is highly relevant that all australopith fifth proximal phalanges exhibit higher values of phalangeal curvature than do any of the extant and fossil *Homo* specimens (Fig. 4), denoting a biological transition in hominins towards less (if any) commitment to arboreal locomotion (at least as it is revealed from manual proximal phalanx anatomy). As with overall phalangeal form (Fig. 3), OH 86 falls exclusively within the modern human range of variation of fifth proximal phalanx curvature (Fig. 4); pooling results for curvature of all non-pollical proximal phalanges place OH 86 once again within the modern human range and in the lowermost range of Gorilla (Supplementary Fig. 5). In addition, compared with the manual phalanges of *O. tugenesis*, *Ar. ramidus*, *Au. afarensis*, *Au. sediba*, the Swartkrans hominins and OH 7—whose powerfully built flexor insertions result in proximal phalanx diaphyseal morphology that includes distinctive, palmarly concave ‘outbowing'—the diaphysis of OH 86 lacks such pronounced flexor insertions and is thus much straighter in medial and lateral views (Fig. 2).

## Discussion

Collectively, these results lead to the conclusion that OH 86 represents a hominin species different from the taxon represented by OH 7, and whose closest form affinities are to modern *H. sapiens* (Fig. 3). However, the geological age of OH 86 obviously precludes its assignment to *H. sapiens*, and ambiguity surrounding the existing potential sample African *H. erectus* (*sensu lato*) hand bones also prohibits its confident assignment to that species at this time. For example, *H. erectus* (*s.l.*) is known from Member 1 of the Swartkrans Formation^37^, which was deposited penecontemporaneously with the formation of the PTK site^38^. However, the co-occurrence of *H. erectus* (*s.l.*) and *P. robustus* at Swartkrans, as well as the lack of any securely associated craniodental and phalanx remains from a single individual at the site, renders taxonomic assignment of the Swartkrans hominin phalanges, at best, tentative (contra, refs 34, 35). The single manual intermediate phalanx of the Kenyan *H. erectus* (*s.l.*) partial skeleton KNM-WT 15,000 possesses a slender, straight and only modestly ridged diaphysis, with trochlear morphology closely approximating the modern human condition (and departing clearly from the condition of australopiths and OH 7)^7^. However, at ∼1.5 Ma, KNM-WT 15,000 is considerably geologically younger than is OH 86. The same holds for the isolated, presumed *H. erectus* (*s.l.*), third metacarpal from the ∼1.42-Ma Kaitio site (Kenya), which possesses, similar to modern humans and *H. neanderthalensis*, a styloid process—related by some to mechanical stability necessary for regular manufacture and use of tools^39^. Further reason for caution in taxonomic assignment of OH 86 is the current lack of spatially and functionally associated hominin craniodental remains from the PTK site.

It is, of course, impossible to reconstruct the whole hand of the OH 86 hominin from what is known of a single phalanx. However, among individual primates, the manual proximal phalanx of one ray assumes similar morphology and relative intrinsic proportions as do that individual's other manual proximal phalanges. This is causing some debate in paleoanthropology when trying to elucidate intrinsic hand proportions in fossil hominins from assemblages of isolated hand elements^4^,^29^,^30^,^40^. Discerning serially homologous phalanges is such a complex task that some studies describing new fossils do not even attempt to assign individual fossil phalanges to a particular ray^28^,^41^, and this is why some scholars designed specific protocols to address this problem^36^, as it is the case of this study (Supplementary Fig. 3). Thus, it is parsimonious to infer that the proximal phalanges of the remaining manual rays of the OH 86 individual were constructed and functioned as did that from the fifth ray of its left hand. From this Occamian perspective, the functional morphology of OH 86 would seem to indicate that the paleoecosystem of Bed I (∼2.0–1.8 Ma) at Olduvai was characterized by the sympatry of a minimum of three distinct hominin species, *P. boisei*, *H. habilis* (*s.l.*) and the OH 86 morph—only the latter of which clearly exhibits phalangeal features indicative of more relaxed postural and locomotive selective pressures on the hand. This hypothesis is harmonious with previous inferences, based on analyses of other anatomical regions, that (contrary to that of *H. erectus s.l.*) the postcranial skeletons of *P. boisei* and/or *H. habilis* reflect significant degrees of arborealism^7^,^13^,^42^,^43^,^44^,^45^. However, because of the mosaic nature of hominid and hominin postcranial evolution (for example, refs 46, 47), the confirmation of lack of arboreal features in the hominin species to which the OH 86 phalanx belonged should await further discovery of more remains from other regions of its hand (and other anatomical regions).

In sum, the complete proximal phalanx reported here demonstrates that just <2 Ma at least one East African hominin taxon/lineage showed marked reduction in manual phalangeal arboreal adaptations (as reflected by the proximal phalanx curvature and flexor sheath ridges development in the shaft), along with the concomitant expression of an overall MHL phalangeal morphology (as far as it is possible to infer from a single phalanx). The skeletons of geologically more recent hominins, who unequivocally possessed MHL hands, also show other important modifications of the postcranium that functioned as a part of a complex adaptive shift to a more fully committed terrestrial life (for example, refs 48, 49, 50, 51). Thus, OH 86 adds to previous ∼1.9–1.8 Ma evidence that indicates that several key aspects of modern human body morphology emerged very early in human evolution. For example, the KNM-ER 3228 hominin pelvis (*cf. H. erectus*, Kenya) resembles those of modern human males^19^, and the Dmanisi postcranial remains (Republic of Georgia) demonstrate that *H. erectus* (*s.l.*) limb proportions were similar to those of modern humans^20^.

The putative presence of a large-bodied, modernly proportioned and modernly capable species of *Homo* (*cf. H. erectus s.l.*) in the early Pleistocene Olduvai basin holds major implications for the potential re-interpretation of traces of hominin behaviour preserved in the numerous Bed I archaeological sites, whose formation has been previously typically attributed to the activities of smaller-bodied, more arboreally adapted *H. habilis*. We are confident that the eventual discovery of more hominin fossils and associated archaeological remains from our on-going fieldwork at the new Olduvai site of PTK will facilitate the detailed investigation of this issue and also shed even more light on the earliest stages of the evolution of the genus *Homo*.

## Methods

### Shape and size analyses

The samples employed for the phalangeal shape/size and included angle analyses are described in Supplementary Tables 2–4. These seven measurements were used to inspect phalangeal size (approximated by the GM) and shape (based on seven Mosimann ratios)^52^,^53^ in our sample (Supplementary Fig. 4). Phalangeal form variation was explored via PCA (Fig. 3a) by including the dimensionless Mosimann ratios and the GM (after log-transformed using natural logarithms). This method, similar to the one described in ref. 54 for geometric data, allows size adjustment of the data while still being able to identify which portions of shape and size contribute to overall phalangeal form variation. Phalangeal form was also summarized using an UPGMA dendrogram on the basis of group centroids (Fig. 3b).

### Phalangeal curvature

Proximal phalanx curvature was estimated using the included angle method described in refs 13, 43, 55. Basically, the included angle (IA) assumes that the shape of the proximal phalanx in lateral view approximates a portion of a circle (see Fig. 4). The radius of curvature (*R*) of the circle is calculated from three measurements: interarticular length (*L*), dorsopalmar midshaft diameter (*D*) and projected height (*H*).


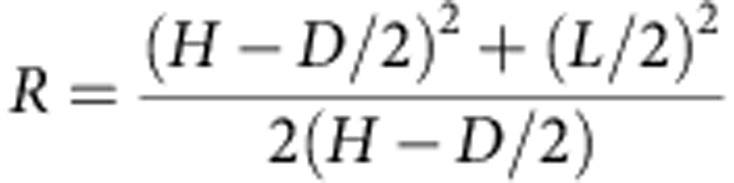


which in turn allows computing the IA


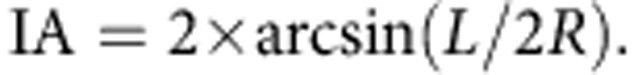


## Additional information

**How to cite this article:** Domínguez-Rodrigo, M. *et al.* Earliest modern human-like hand bone from a new >1.84-million-year-old site at Olduvai in Tanzania. *Nat. Commun.* 6:7987 doi: 10.1038/ncomms8987 (2015).

## Supplementary Material

Supplementary Information Supplementary Figures 1-5, Supplementary Tables 1-5 and Supplementary References

## References

[b1] Napier J. Hands. 180 (Princeton Univ. Press (1993).

[b2] Susman R. L. Fossil evidence for early hominid tool use. Science 265, 1570–1573 (1994). 8079169 10.1126/science.8079169

[b3] Marzke M. W. Precision grips, hand morphology, and tools. Am. J. Phys. Anthropol. 102, 91–110 (1997). 9034041 10.1002/(SICI)1096-8644(199701)102:1<91::AID-AJPA8>3.0.CO;2-G

[b4] Alba D. M., Moyà-Solà S. & Köhler M. Morphological affinities of the *Australopithecus afarensis* hand on the basis of manual proportions and relative thumb length. J. Hum. Evol. 44, 225–254 (2003). 12662944 10.1016/s0047-2484(02)00207-5

[b5] Marzke M. W. in Primate Evolution eds Else J. G., Lee P. C. 203–209Cambridge University Press (1986).

[b6] Skinner M. M. *et al.* Human-like hand use in *Australopithecus africanus*. Science 347, 395–399 (2015). 25613885 10.1126/science.1261735

[b7] Moyà-Solà S., Köhler M., Alba D. M. & Almécija S. Taxonomic attribution of the Olduvai Hominid 7 manual remains and the functional interpretation of hand morphology in robust australopithecines. Folia Primatol. 79, 215–250 (2008). 18277078 10.1159/000113458

[b8] Almécija S., Moyà-Solà S. & Alba D. M. Early origin for human-like precision grasping: a comparative study of pollical distal phalanges in fossil hominins. PLoS ONE 5, e11727 (2010). 20661444 10.1371/journal.pone.0011727 PMC2908684

[b9] Almécija S., Wallace I. J., Judex S., Alba D. M. & Moyà-Solà S. Comment on “Human-like hand use in *Australopithecus africanus*”. Science 348, 1101 (2015). 10.1126/science.aaa8414 26045428

[b10] Semaw S. *et al.* 2.5-million-year-old stone tools from Gona, Ethiopia. Nature 385, 333–336 (1997). 9002516 10.1038/385333a0

[b11] Domínguez-Rodrigo M., Pickering T. R., Semaw S. & Rogers M. J. Cutmarked bones from Pliocene archaeological sites at Gona, Afar, Ethiopia: implications for the function of the world's oldest stone tools. J. Hum. Evol. 48, 109–121 (2005). 15701526 10.1016/j.jhevol.2004.09.004

[b12] Harmand S. *et al.* 3.3-million-year-old stone tools from Lomekwi 3, West Turkana, Kenya. Nature 521, 310–315 (2015). 25993961 10.1038/nature14464

[b13] Stern J. T. Jr. & Susman R. L. The locomotor anatomy of *Australopithecus afarensis*. Am. J. Phys. Anthropol. 60, 279–317 (1983). 6405621 10.1002/ajpa.1330600302

[b14] Kivell T. L., Kibii J. M., Churchill S. E., Schmid P. & Berger L. R. *Australopithecus sediba* hand demonstrates mosaic evolution of locomotor and manipulative abilities. Science 333, 1411–1417 (2011). 21903806 10.1126/science.1202625

[b15] Haile-Selassie Y. *et al.* A new hominin foot from Ethiopia shows multiple Pliocene bipedal adaptations. Nature 483, 565–569 (2012). 22460901 10.1038/nature10922

[b16] DeSilva J. M. *et al.* The lower limb and mechanics of walking in *Australopithecus sediba*. Science 340, 1232999 (2013). 23580534 10.1126/science.1232999

[b17] Lovejoy C. O., Meindl R. S., Ohman J. C., Heiple K. G. & White T. D. The Maka femur and its bearing on the antiquity of human walking: applying contemporary concepts of morphogenesis to the human fossil record. Am. J. Phys. Anthropol. 119, 97–133 (2002). 12237933 10.1002/ajpa.10111

[b18] Ward C. V., Kimbel W. H. & Johanson D. C. Complete fourth metatarsal and arches in the foot of *Australopithecus afarensis*. Science 331, 750–753 (2011). 21311018 10.1126/science.1201463

[b19] Rose M. D. A hominine hip bone, KNM-ER 3228, from East Lake Turkana, Kenya. Am. J. Phys. Anthropol. 63, 371–378 (1984). 6428239 10.1002/ajpa.1330630404

[b20] Lordkipanidze D. *et al.* Postcranial evidence from early *Homo* from Dmanisi, Georgia. Nature 449, 305–310 (2007). 17882214 10.1038/nature06134

[b21] Domínguez-Rodrigo M. *et al.* Disentangling hominin and carnivore activities near a spring at FLK North (Olduvai Gorge, Tanzania). Quatern. Res. 74, 363–375 (2010).

[b22] Deino A. L. ^40^Ar/^39^Ar dating of Bed I, Olduvai Gorge, Tanzania, and the chronology of early Pleistocene climate change. J. Hum. Evol. 63, 251–273 (2012). 22809744 10.1016/j.jhevol.2012.05.004

[b23] Uribelarrea D. *et al.* Geo-archaeological and geometrically corrected reconstruction of the 1.84 Ma FLK Zinj paleolandscape at Olduvai Gorge, Tanzania. Quatern. Int. 322-323, 7–31 (2014).

[b24] Gommery D. & Senut B. La phalange distale du pouce d'*Orrorin tugenensis* (Miocène supérieur du Kenya). Geobios 39, 372–384 (2006).

[b25] Shrewsbury M. M., Marzke M. W., Linscheid R. L. & Reece S. P. Comparative morphology of the pollical distal phalanx. Am. J. Phys. Anthropol. 121, 30–47 (2003). 12687581 10.1002/ajpa.10192

[b26] Lovejoy C. O., Simpson S. W., White T. D., Asfaw B. & Suwa G. Careful climbing in the Miocene: the forelimbs of *Ardipithecus ramidus* and humans are primitive. Science 326, 70–708 (2009). 19810196

[b27] Almécija S., Smaers J. B. & Jungers W. L. The evolution of human and ape hand proportions. Nat. Commun. 6, 7717 (2015). 26171589 10.1038/ncomms8717 PMC4510966

[b28] Ward C. V., Kimbel W. H., Harmon E. H. & Johanson D. C. New postcranial fossils of *Australopithecus afarensis* from Hadar, Ethiopia (1990-2007). J. Hum. Evol. 63, 1–51 (2012). 22652491 10.1016/j.jhevol.2011.11.012

[b29] Almécija S. & Alba D. M. On manual proportions and pad-to-pad precision grasping in *Australopithecus afarensis*. J. Hum. Evol. 73, 88–92 (2014). 24636370 10.1016/j.jhevol.2014.02.006

[b30] Rolian C. & Gordon A. D. Reassessing manual proportions in *Australopithecus afarensis*. Am. J. Phys. Anthropol. 152, 393–406 (2013). 24104947 10.1002/ajpa.22365

[b31] Kivell T. L. A comparative analysis of the hominin triquetrum (SKX 3498) from Swartkrans, South Africa. S. Afr. J. Sci. 107, 60–69 (2011).

[b32] Napier J. Fossil hand bones from Olduvai Gorge. Nature 196, 409–411 (1962).

[b33] Leakey L. S. B., Tobias P. V. & Napier J. R. A new species of the genus *Homo* from Olduvai Gorge. Nature 202, 7–9 (1964). 14166722 10.1038/202007a0

[b34] Susman R. L. Hand of *Paranthopus robustus* from member 1, Swartkrans: fossil evidence for tool behavior. Science 240, 781–784 (1988). 3129783 10.1126/science.3129783

[b35] Susman R. L. in The Evolutionary History of the Robust Australopithecines ed. Grine F.E. 133–148Aldine de Gruyter (1988).

[b36] Garrido Varas C. E. & Thompson T. J. U. Metric dimensions of the proximal phalanges of the human hand and their relationship to side, position, and asymmetry. HOMO 62, 126–143 (2011). 21168843 10.1016/j.jchb.2010.07.005

[b37] Broom R. & Robinson J. T. Man contemporaneous with the Sawartkrans ape-man. Am. J. Phys. Anthropol. 8, 151–156 (1950). 15432627 10.1002/ajpa.1330080211

[b38] Gibbon R. J. *et al.* Cosmogenic nuclide burial dating of hominin-bearing Pleistocene cave deposits at Swartkrans, South Africa. Quat. Geochronol. 24, 10–15 (2014).

[b39] Ward C. V., Tocheri M. W., Plavcan J. M., Brown F. H. & Manthi F. K. Early Pleistocene third metacarpal from Kenya and the evolution of modern human-like hand morphology. Proc. Natl Acad. Sci. USA 111, 121–124 (2014). 24344276 10.1073/pnas.1316014110 PMC3890866

[b40] Rolian C. & Gordon A. D. Response to Almécija and Alba (2014) – On manual proportions in *Australopithecus afarensis*. J. Hum. Evol. 73, 93–97 (2014). 24957903 10.1016/j.jhevol.2014.05.002

[b41] Bush M. E., Lovejoy C. O., Johanson D. C. & Coppens Y. Hominid carpal, metacarpal, and phalangeal bones recovered from the Hadar Formation: 1974-1977 collections. Am. J. Phys. Anthropol. 57, 651–677 (1982).

[b42] Susman R. L. & Stern J. T. Functional morphology of *Homo habilis*. Science 217, 931–934 (1982). 17747955 10.1126/science.217.4563.931

[b43] Susman R. L., Stern J. T. J. & Jungers W. L. Arboreality and bipedality in the Hadar hominids. Folia Primatol. 43, 113–156 (1984). 6440837 10.1159/000156176

[b44] Johanson D. C. *et al.* New partial skeleton of *Homo habilis* from Olduvai Gorge, Tanzania. Nature 327, 205–209 (1987). 3106831 10.1038/327205a0

[b45] Domínguez-Rodrigo M. *et al.* First partial skeleton of a 1.34-million-year-old *Paranthropus boisei* from Bed II, Olduvai Gorge, Tanzania. PLoS ONE 8, e80347 (2013). 24339873 10.1371/journal.pone.0080347 PMC3855051

[b46] Almécija S., Alba D. M., Moyà-Solà S. & Köhler M. Orang-like manual adaptations in the fossil hominoid *Hispanopithecus laietanus*: first steps towards great ape suspensory behaviours. Proc. R. Soc. B. 274, 2375–2384 (2007). 10.1098/rspb.2007.0750 PMC2274979 17623642

[b47] Almécija S. *et al.* The femur of *Orrorin tugenensis* exhibits morphometric affinities with both Miocene apes and later hominins. Nat. Commun. 4, 2888 (2013). 24301078 10.1038/ncomms3888

[b48] Carretero J. M., Arsuaga J. L. & Lorenzo C. Clavicles, scapulae and humeri from the Sima de los Huesos site (Sierra de Atapuerca, Spain). J. Hum. Evol. 33, 357–408 (1997). 9300347 10.1006/jhev.1997.0128

[b49] Lorenzo C., Arsuaga J. L. & Carretero J. M. Hand and foot remains from the Gran Dolina Early Pleistocene site (Sierra de Atapuerca, Spain). J. Hum. Evol. 37, 501–522 (1999). 10496998 10.1006/jhev.1999.0341

[b50] Arsuaga J. L. *et al.* A complete human pelvis from the Middle Pleistocene of Spain. Nature 399, 255–258 (1999). 10353247 10.1038/20430

[b51] Carretero J. M., Lorenzo C. & Arsuaga J. L. Axial and apendicular skeleton of *Homo antecessor*. J. Hum. Evol. 37, 459–499 (1999). 10496997 10.1006/jhev.1999.0342

[b52] Mosimann J. E. Size allometry: size and shape variables with characterizations of the lognormal and generalized gamma distributions. J. Am. Stat. Assoc. 65, 930–945 (1970).

[b53] Jungers W. L., Falsetti A. B. & Wall C. E. Shape, relative size, and size-adjustments in morphometrics. Yearb. Phys. Anthropol. 38, 137–161 (1995).

[b54] Mitteroecker P., Gunz P., Bernhard M., Schaefer K. & Bookstein F. L. Comparison of cranial ontogenetic trajectories among great apes and humans. J. Hum. Evol. 46, 679–698 (2004). 15183670 10.1016/j.jhevol.2004.03.006

[b55] Stern J. T., Jungers W. L. & Susman R. Quantifying phalangeal curvature: an empirical comparison of alternative methods. Am. J. Phys. Anthropol. 97, 1–10 (1995). 7645670 10.1002/ajpa.1330970102

[b56] Lorenzo C. *et al.* Early Pleistocene human hand phalanx from the Sima del Elefante (TE) cave site in Sierra de Atapuerca (Spain). J. Hum. Evol. 78, 114–121 (2015). 25200886 10.1016/j.jhevol.2014.08.007

