# Supplementary material for: Earliest modern human-like hand bone from a new >1.84-million-year-old site at Olduvai in Tanzania
Source: Nat Commun. 2015 Aug 18;6:7987. doi: 10.1038/ncomms8987 (PMC4557276; doi:10.1038/ncomms8987)
Supplement: Supplementary Information — Supplementary Figures 1-5, Supplementary Tables 1-5 and Supplementary References [file ncomms8987-s1.pdf]

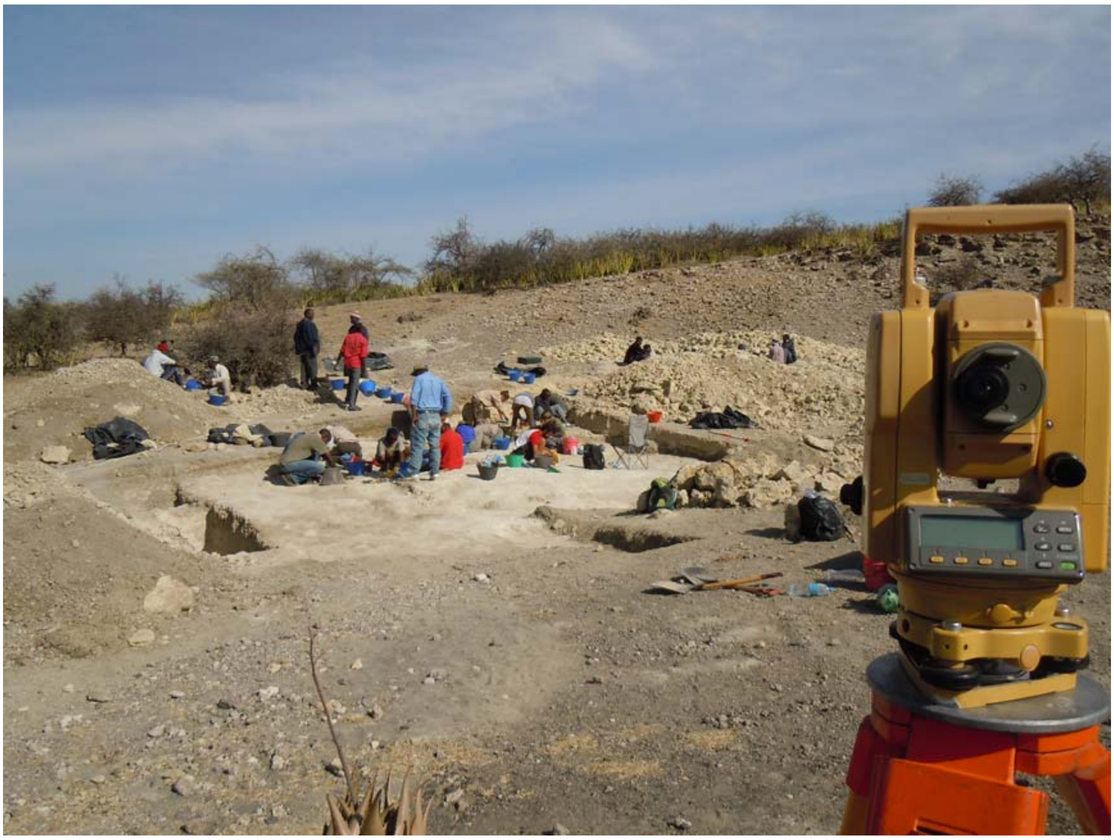

Supplementary Figure 1. Excavation of the Phillip Tobias Korongo (PTK) site by The Olduvai Paleoanthropology and Paleoecology Project (TOPPP). Informal view to the west side of the site in 2013.

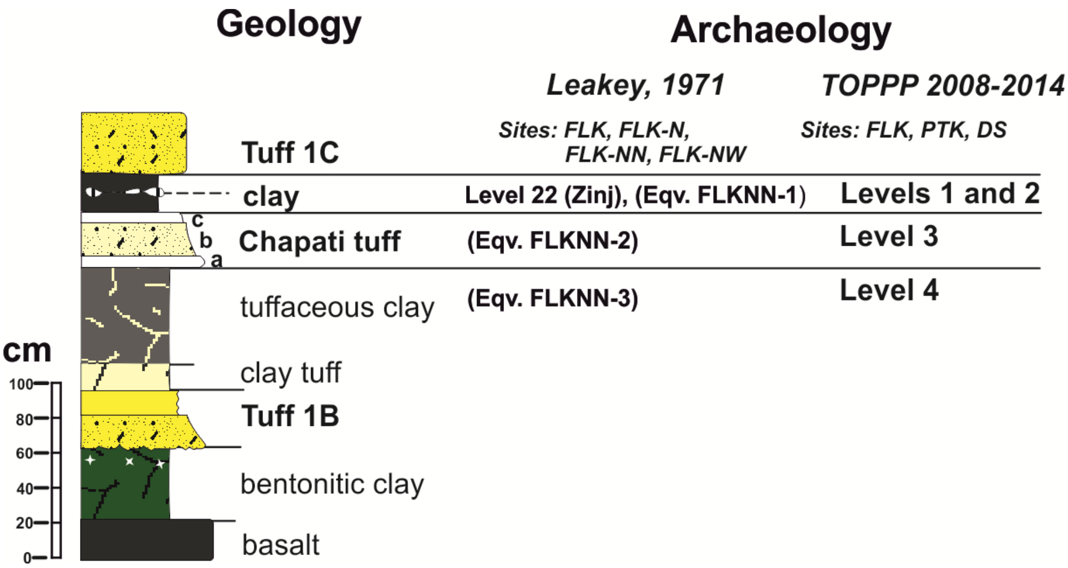

Supplementary Figure 2. Stratigraphic section of Lower and Middle Bed I (Olduvai Formation, Tanzania) and location of the PTK site. Archaeological level 3, which yielded the new OH 86 hominin proximal phalanx fossil, is situated stratigraphically in Layer C of the Chapati Tuff.

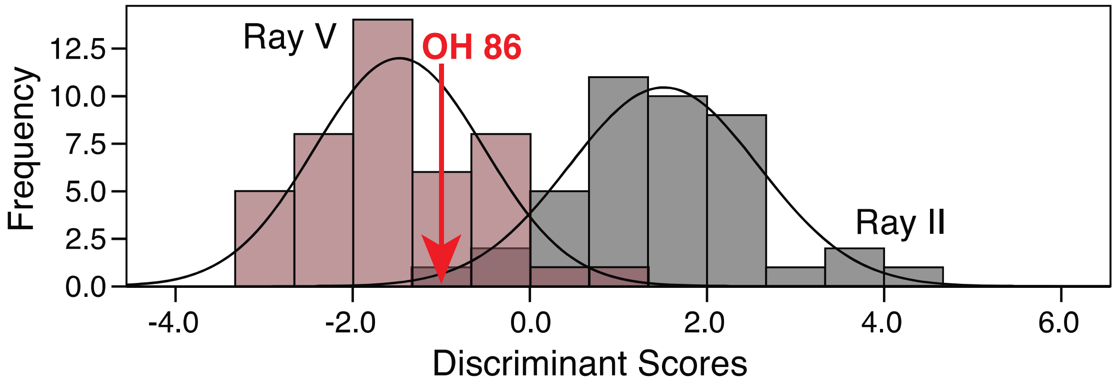

Supplementary Figure 3. Discriminant analysis of human proximal phalanges II and V from the *Homo sapiens* sample compared to the OH 86 fossil. OH 86 most probably belongs to ray V. The histogram shows the discriminant function scores of an analysis carried out on the original (seven) measurements, which yielded better results than size-adjusting the data. Ninety-four per cent of the original cases are correctly classified, and 91.8% of the cross-validated cases are correctly classified. OH 86 is classified as belonging to ray V, with a much higher probability than belonging to ray II (0.953 vs. 0.047 respectively; using Fisher classification coefficients, also in all cases below). The other isolated fossil hominin specimens included in the analyses were also classified as fifth proximal phalanges with the following probabilities (V vs. II): AL333-62 (p = 0.972 vs. p = 0.028), StW 28 (p = 0.999 vs. p = 0.001), ATE9-2 (p = 0.9 vs. p = 0.1). The modern human sample includes 40 individuals (for both rays).

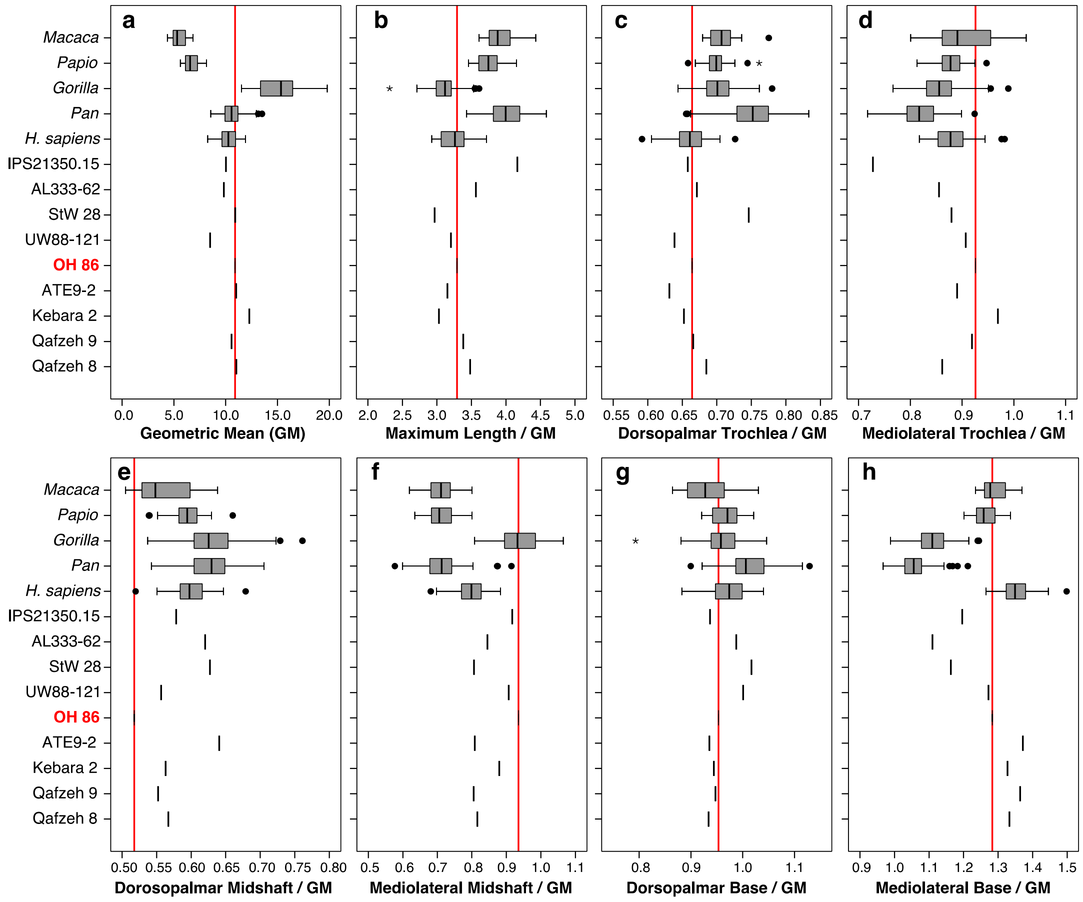

Supplementary Figure 4. Size and Mosimann shape ratios of proximal phalanges of ray V. Boxes represent 25th and 75th percentiles, centerline is the median, whiskers represent non-outlier range, dots are outliers, and asterisks are extreme outliers. The position of OH 86 is extended onto the remaining taxa (red line) to facilitate comparisons. The plesiomorphic Miocene great ape, *Pierolapithecus catalaunicus* (IPS21350; ~12 Ma), is included in all analyses to give a sense of evolutionary polarity. Extant samples for each boxplot are *Homo sapiens* (n = 40), *Pan* (n = 82), *Gorilla* (n = 108), *Papio* (n = 34), and *Macaca* (n = 18). Fossil proveniences are indicated in Supplementary Table 2.

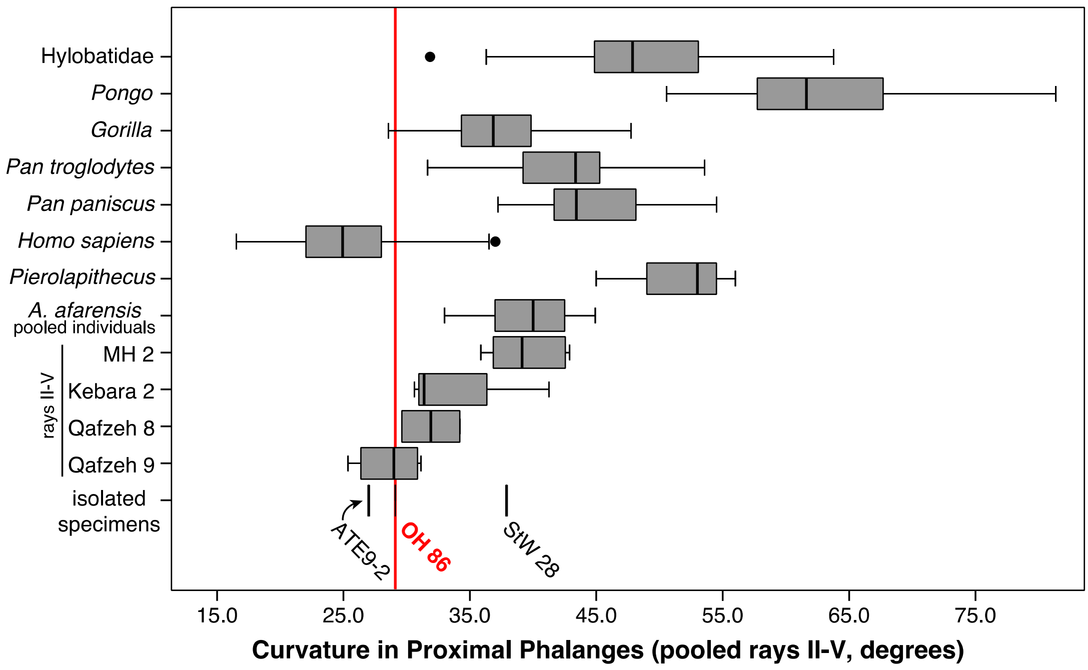

Supplementary Figure 5. Phalangeal curvature in extant and fossil hominoids (pooling rays II-V). Included angle values (in degrees) in modern and fossil samples of proximal phalanges. The position of OH 86 is extended onto the remaining taxa (red line) to facilitate comparisons. Even though pooling the curvature of rays II to V increases the overlapping ranges between each taxa, OH 86 is still within the modern human variation (distinct from australopiths) and the lowermost range of gorillas. Boxes represent 25th and 75th percentiles, centerline is the median, whiskers represent non-outlier range, and the dots are outliers. Extant samples for each boxplot are *Homo sapiens* (n = 146), *Pan paniscus* (n = 38), *Pan troglodytes* (n = 63), *Gorilla* (n = 88), *Pongo* (n = 68), and Hylobatidae (n = 88). Fossil proveniences are indicated in Supplementary Table 4.

Supplementary Table 1. Measurements of OH 86. All linear dimensions refer to maximum diameters (in mm) and the included angle is in degrees.

Maximum Length	35.9
Dorsopalmar Trochlea	6.7
Mediolateral Trochlea	10.1
Dorsopalmar Midshaft	5.1
Mediolateral Midshaft	10.2
Dorsopalmar Base	10.4
Mediolateral Base	14.0
Included Angle	29.1

Supplementary Table 2. Extant sample for the shape analyses (discriminant function, Mosimann ratios and principal components analysis).

		N
Homo	*sapiens*[a]	40
Pan	*troglodytes*[b]	62
	paniscus[c]	20
Gorilla	*gorilla*[d]	48
	beringei[e]	40
	unknown[f]	20
Papio	*hamadryas*[g]	34
Macaca	*fascicularis*[h]	3
	fuscata[h]	2
	maura[h]	1
	nemestrina[i]	5
	nigra[h]	2
	silenus[h]	2
	sinica[h]	1
	sylvanus[h]	2
**Total sample**		**282**

Superscripts indicate the collection provenience for each taxon. (a) UAB, CMNH, SBU, Naturalis; (b) AMNH, RMCA, USNM, MCZ, Naturalis, SBU; (c) RMCA, AMNH, MCZ, SBU; (d) AMNH, RMCA, CMNH, MCZ, Naturalis; (e) RMCA, AMNH, USNM, MCZ; (f) AMNH; (g) AMNH, Naturalis, SBU; (h) Naturalis; (i) Naturalis, MCZ. Abbreviations: UAB (Universitat Autònoma de Barcelona), CMNH (Cleveland Museum of Natural History), SBU (Stony Brook University), Naturalis (Naturalis Biodiversity Center), AMNH (American Museum of Natural History), RMCA (Royal Museum of Central Africa), USNM (National Museum of Natural History), MCZ (Museum of Comparative Zoology).

Supplementary Table 3. Extant sample for included angle per ray (total phalanges in sample = 491).

		II	III	IV	V
Homo	*sapiens*	36	37	37	36
Pan	*troglodytes*	15	16	16	16
	paniscus	10	10	10	8
Gorilla	sp.	22	22	22	22
Pongo	sp.	17	18	17	16
Symphalangus	*syndactylus*	5	5	5	5
Hylobates	sp.	17	17	17	17
**Total samples**		**122**	**125**	**124**	**120**

The original curvature data were kindly provided by Jack Stern and Randy Susman, details on the sample composition and provenience are provided in ref. 6.

Supplementary Table 4. Fossil specimens, that have been attributed to the fifth ray, included in this study.

	species	data source
IPS21350.15	*Pierolapithecus catalaunicus*	Almécija et al., 2009[1]
AL333-62	*Australopithecus afarensis*	Bush et al., 1982[2]
Stw 28	*Australopithecus* sp.	Lorenzo et al., 2015[3]
UW88-121	*Australopithecus sediba*	Kivell et al., 2011[4]
ATE9-2	*Homo* sp.	Lorenzo et al., 2015[3]
OH86	*Homo* sp.	this study
Kebara 2	*Homo neanderthalensis*	this study
Qafzeh 8	early *Homo sapiens*	this study
Qafzeh 9	early *Homo sapiens*	this study

Supplementary Table 5. Results of the principal components analysis of phalangeal form.

	**Principal Component 1**	**Principal Component 2**
% variance	77.229	12.876
Maximum Length / GM	**-0.72**	**0.628**
Dorsopalmar Trochlea / GM	-0.042	**0.731**
Mediolateral Trochlea / GM	-0.203	**-0.521**
Dorsopalmar Midshaft / GM	0.442	0.317
Mediolateral Midshaft / GM	**0.804**	-0.457
Dorsopalmar Base / GM	-0.032	**0.511**
Mediolateral Base / GM	-0.417	**-0.777**
GM (geometric mean)	**0.992**	0.100

Each variable was size log-transformed (using natural logarithm) prior to inclusion into the analysis. Absolute loading ≥ 0.5 are marked in bold. Only the two first axes provided meaningful discrimination among extant group.

Supplementary References

1 Almécija, S., Alba, D. M. & Moyà-Solà, S. *Pierolapithecus* and the functional morphology of Miocene ape hand phalanges: paleobiological and evolutionary implications. *J. Hum. Evol.* **57**, 284-297, (2009).

2 Bush, M. E., Lovejoy, C. O., Johanson, D. C. & Coppens, Y. Hominid carpal, metacarpal, and phalangeal bones recovered from the Hadar Formation: 1974-1977 collections. *Am. J. Phys. Anthropol.* **57**, 651-677, (1982).

3 Lorenzo, C. *et al.* Early Pleistocene human hand phalanx from the Sima del Elefante (TE) cave site in Sierra de Atapuerca (Spain). *J. Hum. Evol.* **78**, 114-121, (2015).

4 Kivell, T. L., Kibii, J. M., Churchill, S. E., Schmid, P. & Berger, L. R. *Australopithecus sediba* hand demonstrates mosaic evolution of locomotor and manipulative abilities. *Science* **333**, 1411-1417, (2011).
